# Lectures on Subjects Connected with Clinical Medicine, Comprising Diseases of the Heart

**Published:** 1847-04-01

**Authors:** 


I. Lectures on Subjects connected with Clinical Medicine, comprising Diseases of the Heart. By *P. M. Latham*, M.D. Physician Extraordinary to the Queen, and late Physician of St. Bartholomew's Hospital. Vol. II. pp. 419. London: Longman and Co., 1846.

[Second Notice.]

II. Traité de Nosographie Medicale. Par *J. Bouillaud*, Professeur de Clinique Medicale à la Faculté de Medecine de Paris, &c. &c. 5 Tomes. 8vo. 1846. Bailliere.

[Second Notice.]

In closing our notice of Dr. Latham's Lectures in our last Number, we intimated our intention of resuming it, so that we might have an opportunity of comparing his sentiments on some of the more important points in the history of Cardiac Diseases with those professed by certain cotemporaneous writers of the French School. We have selected M. Bouillaud as the representative of Continental Medicine upon this occasion, as being an author of unquestionably no ordinary talent, and one who has devoted his especial attention to the class of disorders in question, and contributed not a little to their more complete elucidation. That he has great and manifold faults as a medical writer cannot be unknown to our readers. In some respects, he is one of the most dangerous guides that could be selected by any young practitioner. Bold, confident, and presumptuous, he labours with most assiduous energy to give currency to his opinions, which all savour of a spirit of extravagance and exaggeration alike in matters of doctrine and in those of practice. He is ever seeking to bring within the domain of arithmetic exactitude the phenomena and course of diseases on the one hand, and to establish with an almost unvarying precision the indications for their therapeutic treatment on the other; as if the former could be weighed in a balance, and the latter could be measured with a plumb-line! Hence it is that he is continually laying down, in the most dogmatic and peremptory tone, certain rules for the detection and cure of most maladies, as if he were describing a mere problem in mathematics to be worked out.

In our former notice of his present work,* we exposed the numerous errors and inconsistencies into which he had fallen in discussing the difficult question of Fevers, and we pointed out the pernicious consequences of medical practitioners allowing themselves to be guided by him in the treatment of this truly multiform class of diseases. It is unnecessary to revert to this subject further than to remind our readers that he actually treats of Synocha or sthenic fever under the terms of Angioitis or inflammation of the vascular system; as we shall have occasion to allude, although very briefly, to the history of genuine Arteritis towards the close of the present article. What often adds much to the difficulty of clearly ascertaining his views, is the strange and most perplexing arrangement of diseases which he has chalked out; so that the reader is obliged to be continually turning from one to another of his five bulky volumes to discover the complete history of any one set of diseases. On no subject is this vexatious confusion more annoying than in his account of Diseases of the Heart. The reader must therefore make some allowance, if the extracts, &c. to be given prove less interesting than we could have desired.

The point, at which we had left off in our analysis of Dr. Latham's valuable work, was where he was treating of what he calls "mock Hypertrophy of the Heart;" in other words, of those cases where the actions of this vital organ are so forcible and impetuous as very naturally to suggest the idea of the actual enlargement of its volume, although in truth no such serious change has taken place.

"Impulse of the heart," says he, "taken alone, however great and however extensive it may be, is not a sure physical sign of hypertrophy. Hypertrophy indeed cannot exist without excess of impulse, but excess of impulse can exist without hypertrophy. When the impulse of the heart is excessive, and at the same time its sounds are obtuse, muffled and indistinct, and the præcordial region presents a larger space than natural which is dull to percussion, then the signs of hypertrophy are complete. And hypertrophy so sure and unquestionable was never cured within my experience. But when the impulse of the heart is in excess, and at the same time its sounds are as loud and clear as ever, or louder and clearer still, and the

---

* *Vide* Medico-Chirurgical Review for July, 1846.

whole præcordial region is quite resonant to percussion save the small space which is naturally dull, then the signs of hypertrophy are incomplete." P. 249.

It is well known that those of mock or simulated Hypertrophy are especially common in young females affected with Chlorosis. We shall therefore first allude to the cardiac, &c. complications of this very frequent disorder, taking the " Nosographie" as our text-book on the occasion.

After alluding to the aspect and general appearance of chlorotic patients which M. Bouillaud, with his accustomed exaggeration, declares to be invariably so marked and decisive that there never ought to be any room for doubt on the part of the physician, he treats of those diagnostic signs which are furnished by the Auscultation of the Heart and Arteries, and by the exploration of the pulse.

" In every well-marked case," says he, " of Chlorosis or Chloro-anæmia, the carotid and subclavian, sometimes also the crural, arteries give out the continuous blowing, humming, or whistling sound, which I have described at length in my treatise on Diseases of the Heart. Not a day passes without my seeing cases of this sort. It is of the highest importance in a diagnostic and therapeutic point of view to ascertain whether this symptom be present or not; and yet it is strangely neglected by a vast number of medical practitioners. For if this one symptom be present, we may confidently assert, from this circumstance alone, that a chlorotic or anæmic condition of the system is present; and, *vice versa*, whenever a patient exhibits the external features of this condition, we may predict that the arterial sound in question will be found."

It is thus described:

" In well-marked Chlorosis may be heard, during the first or systolic action of the heart's movements (au premier temps des mouvements du cœur), a gentle, soft, and silky blowing sound; it is most distinct over the orifice of the aorta, and extends along the course of this vessel to where it gives off the carotids and subclavians, with whose continuous murmur it becomes confounded.

" The pulse of chlorotic patients is soft, unresisting, and conveys to the finger, when the artery is gently pressed, a sort of fremitus, with large vibrations, of the column of blood; it may be called the ' chlorotic fremitus,' to distinguish it from other vibratory movements present in certain organic diseases of the heart and blood-vessels. It is exactly similar to the sensation felt by the finger, when this is applied to the canal of the urethra during the act of micturition. The touch alone serves to satisfy us that the artery is not sufficiently full, and that the blood, contained within it, has not its due consistence and density. In cases of simple anæmia, the touch will also enable us to ascertain that the volume or calibre of the vessel is less than in the healthy condition."

According to M. Bouillaud, the Diagnosis of the Cardiac affections, dependent upon an attenuated state of the blood, is yet most imperfectly understood among his fellow-countrymen.

" During the last fifteen years that my attention has been directed in an especial manner to the history of chlorotic and anæmic maladies, scarcely a day has passed over that I have not seen cases where they have been mistaken, and confounded, either with inflammatory or with organic diseases by the most distinguished physicians of the different schools, which have hitherto disputed the empire of medicine;—some, diagnosticating inflammatory or organic diseases where all was dependent upon a chlorotic condition; while others, on the contrary, have mistaken cases of the most serious and decided organic alteration for those arising from mere impoverishment and attenuation of the circulating fluid.

I do not exaggerate when I affirm that errors of this sort are, in the present day, committed by thousands in different countries of medical Europe. What is the cause of this? Because, in the schools to which we allude, observation has not been directed to the study of the morbid changes of the fluids, of the blood more particularly, and because the exact methods of exploration—those which furnish the physical and characteristic signs of most diseases—have not been duly practised. So true is this that, among the several hundred of pupils who for some years past have followed my clinical practice, there is not one who, after a few months' assiduous study, has not learned to discriminate with accuracy every case of Chlorosis and Anæmia. All have been astonished to see almost daily patients come into our wards, labouring under affections which had been generally treated by antiphlogistic remedies in consequence of having been mistaken for an inflammatory or congestive malady, or for some organic lesion, such as Aneurism, Hypertrophy of the Heart, Scirrhus or Cancer of the Liver, Spleen, Stomach, &c."

M. Bouillaud then adds:—

"The *physical* signs of chlorosis and of chloro-anæmia are so manifest and so sure that for a physician to be unable to distinguish them, must imply on his part an utter ignorance of this branch of semeiology."

He is forced however to admit that, in certain instances, the diagnosis is by no means without some difficulty.

"In those cases, more numerous than is generally believed, where Chlorosis, Anæmia, and their nervous accompaniments, coincide with an organic affection either of the Heart or some other viscus, the physician will be able to recognise the different elements of these complex cases by means of the signs or symptoms proper to each of them. But it must be confessed that these are problems, the solution of which can be made only by those who have been long familiar with all the difficulties of exact clinical medicine, and the number of whom is unfortunately not so large as it ought to be. Those, who have followed our practice, know full well that examples of the most varied morbid complications are not of unfrequent occurrence, and that, nevertheless, by means of a minute and searching examination, it rarely happens that the diagnosis is not determined with the greatest exactitude. But, if physicians shall still persist in taking Chlorosis for a chronic metritis, or for an asthenia of the generative organs, or for a chronic arteritis,* or for an asthenic condition of the sanguineous system, consisting principally in a diminution of the stimulant properties of the blood, or for a chronic gastritis, or for an aneurism of the Heart, &c.; if, I say, these and such-like errors continue to be committed by medical practitioners, then assuredly the fault lies not so much in the art of diagnosis, as in the culpable ignorance of him who practises it: *non crimen artis, quod professoris est.*"

It will be perceived that, in the above extracts from M. Bouillaud's description of the auscultatory phenomena of Chlorosis and other kindred maladies, he makes the arteries the seat of the continuous humming sound, the "bruit de diable," (as he has called it,) now recognised as one of the surest signs of an anæmic condition of the system, or, perhaps we

* This strange ætiological doctrine has been much insisted on by the celebrated Italian professor, Tommassini. We exposed the absurdity of the hypothesis in the number of this Journal for January 1845. M. Bouillaud may well express his astonishment that any sensible man should entertain such an idea; for there is surely not a shadow of probability that can be adduced in its favour.

should rather say, of a poor and watery state of the blood. The omission of even so much as a passing allusion to any other interpretation of the phenomenon in question is not very creditable either to his professional knowledge or to his professional candour. Let us see what Dr. Latham has said upon the subject. After alluding to the blowing or bellows-sound heard over the heart and the *trajet* of the large arteries in chlorosis, he goes on to remark:—

"Generally accompanying the endocardial and arterial murmur, when it is owing to anæmia or an impoverished blood, there is another sound quite different in kind, and formed neither in the heart nor in the arteries, but traceable to the same pathological condition.

"In following the murmur from the heart along the aorta and the subclavian artery, and then above the clavicle, when you reach the carotid you find a new sound superadded to it. You perceive the bellows-murmur coming and going with distinct whiffs, and keeping time with the systole of the heart in the neck as in the chest; but in the neck you perceive, moreover, *a continuous hum*, like that which reaches the ear from the hollow of a marine shell. This is a thing so evident, that it was noticed and described, and variously speculated upon by those, who first practised auscultation. But their speculations were wide of the mark. Whence or how it arose no one could tell, until the sagacity of Dr. Ogier Ward traced it to the veins, and showed it to proceed from the movement of the blood within them.

"The vein, which offers itself most readily to the application of the stethoscope, and admits all the easy experiments which serve to certify the fact, is the internal jugular. Place the instrument upon the neck by the side of the trachea, and pretty close to it, and at the same time rest your finger upon the space between the angle of the jaw and the mastoid process; and when your ear has caught a continuous humming sound, and listened for a while and made sure of it, then press your finger firmly down upon the vein, and the sound, if it be the true venous murmur, will immediately cease; then raise your finger, and, if it be the true venous murmur, it will immediately return.

"A little management and address are needed, to find this venous murmur, and then keep it within hearing when you have found it. I have seen it found by accident, heard for a minute, and then lost and never heard again. The instrument has been laid carelessly upon the neck and the murmur has been audible immediately; and then, in expectation of making it heard to more advantage, the neck has been put upon the stretch, the chin raised and the head thrown back, or turned far round to the opposite side, whereupon the murmur has ceased. Then the neck has been relaxed, the head brought forward, and the chin inclined towards the sternum, but the murmur has not returned. The truth is, a very free current of blood is essential to the production of the venous murmur. A slight degree of pressure upon the vein will alter its character, and pressure very far short of that which would arrest the current of blood will abolish it altogether. And thus, the neck being put upon the stretch, the muscles, which lie parallel with the vein and across it, are made to exercise pressure enough upon it to interfere with the free current of blood, and to stop the sound; or the neck being relaxed, the vein and the integuments get folded together, and so pressure is produced in another way, and this equally stops the sound. Try different degrees of pressure upon the internal jugular vein with the stethoscope when the venous murmur is distinctly audible, and you will find how lightly you must hold the instrument to keep it constantly within hearing, how inconsiderable an amount of pressure will obliterate it, and how each degree short of that which obliterates it will give it sundry varieties, and make it musical." Vol. I. p. 72.

The circumstances connected with the history of this Venous Murmur have not yet, it must be confessed, been accurately made out. We have just seen that Dr. Latham states that the slightest tension of the muscles of that side of the neck, where the sound is heard, will generally cause it to cease; whereas, other auscultators assert, as the result of their experience, that "it is augmented when the vein and sterno-mastoid muscle are made tense, by turning the head strongly to the side opposite to that which you are examining." Our observation leads us rather to side with Dr. Latham. The pressure of the stethoscope must be steady, but not very strong, to catch the murmur. We have detected it much oftener on the right than on the left side of the neck. Occasionally it may be heard on both sides, but generally upon the right one most distinctly. It is sometimes not easy to recognise the venous murmur at once, in consequence of the marked blowing or whiffing to be heard in the subclavian and carotid arteries at the same time, especially too as the impulse or throb of these vessels is also stronger than usual. As we remarked upon a former occasion, the venous sound has more resembled, to our hearing, that produced by water rushing along a leaden pipe than any other sound that we know. It is, no doubt, a most valuable diagnostic phenomenon; as its presence, when once clearly made out, will go very far to determine the real nature of many cases—in male, as well as in female, patients—where the symptoms so closely resemble those of decided organic disease of the heart, that even the most experienced may hesitate at once to decide.

We need scarcely say that, on the accuracy of Diagnosis, will entirely depend the appropriateness and success of the Treatment that will be recommended. The mistakes, that are so often committed in the discrimination of the real nature of many cases of cardiac disease, are alike discreditable to our profession and most pernicious to our patients. If the case be one of chlorotic or anæmic disturbance of the heart's actions, every one is acquainted with the remedies that will almost infallibly bring about a cure; and should it be one of organic lesion of that vital organ, we shall still have the satisfaction of knowing that, by judicious treatment, a most essential mitigation of suffering may be insured to the poor sufferer.

Before alluding to the general principles of treatment, as laid down by Dr. Latham, in actual hypertrophic enlargement of the Heart, let us briefly notice what M. Bouillaud has said on the important subject of its ætiology. The following passage in his fourth volume will best exhibit his views on this point.

"If the general law, which we have propounded in our remarks upon the *rationale* of Hypertrophy,* be not an empty hypothesis, it may be fairly presumed that hypertrophy of the muscular substance of the Heart ought to be frequently met with after long-continued inflammation of its lining membranes, more especially of its inner one, or, in other words, of the Endocardium. The results of

---

* "All accurate observers know how frequent an occurrence it is to meet with hypertrophic thickening in organs, which have been the seat of a long-continued inflammatory congestion. And the remarkable circumstance is that, in the cases alluded to, the pure and simple Hypertrophy implicates the tissues which are adjacent to where the inflammation was seated, more than the tissue itself that was immediately affected. (This becomes enlarged, it is true; but then it almost invariably experiences, at the same time, some serious alteration, such as softening, induration, disorganization, &c.) Thus it is that the cellular observation, in 500 cases at least, have convinced me of the truth of this position, which may now, therefore, be regarded as an established general fact or law in cardiac pathology. It is not to be supposed from this that we dispute the agency of other causes in the production of this disease. Those enumerated by Corvisart—'contraction of the arteries, or loss of balance between the calibre of the vessels and the quality of blood to be sent through them; all the obstacles to the circulation of the blood, whether arising from a vice of organisation, from any pathological condition, from the influence of moral emotions, or from corporeal efforts or exercises; perhaps, also, the more or less stimulant quality of the blood itself, exciting the organ to more or less energetic contraction'—have unquestionably a morbific effect. One of the most curious and instructive illustrations of one of these agencies, enumerated by Corvisart, may be found in what is apt to take place in the right cavities of the heart in persons in whom the *foramen Botallii* remains open. The principal, if not the only, cause of the hypertrophy of the right ventricle (whose walls have been known to become nearly an inch and a half in thickness), under such circumstances, appears to be the admixture of a certain portion of arterial—which is more stimulating—with the venous blood within its cavity. This opinion seems to be confirmed by what we find to take place in a vein, between which and its adjacent artery a communication has been accidentally established; it becomes thickened, hypertrophied, and, if we may so say, *arterialised.* Now, the right ventricle of the heart may be fairly considered to be analogous to a vein adherent to a sort of artery, represented by the left ventricle. The *venous* heart, therefore, becomes hypertrophied or *arterialised,* just as a vein does where its contents communicate with those of an artery. So striking, indeed, is the resemblance of the right to the left ventricle in the cases alluded to, that several of the older writers, and Corvisart is among the number, have fancied that there was a sort of transposition of the ventricles. Morgagni, in his report of one such case, has expressly said:—*ventriculus sinister formâ erat quâ erat dexter, et dexter vicissim quâ sinister; et quanquam hoc latior, parietibus tamen crassioribus.*

"If the explanation which we propose be just, the conjecture of Corvisart, 'that the more or less stimulant qualities of the blood may perhaps be one of the exciting causes of hypertrophy of the heart,' would assume the importance of a demonstrated truth. We must not however omit to take into account other causes, which may act in concert with that one now suggested."

Supposing, now, that the nature of the cardiac disease has been clearly made out, and that no reasonable doubt can be entertained that an actual enlargement of the heart exists, the next questions for the physician come to be, what is the prognosis that he is to form of the case? and what is the amount or degree of benefit that he may justly expect from the resources of his art?

According to Dr. Latham's experience, genuine and absolute Hypertrophy of the heart—as ascertained in the manner which has been

---

tissue and the lymphatic glands usually become hypertrophied after chronic ulcerations of the skin and mucous membranes, that the fibrous textures of the joints and the articular extremities of the bones themselves become enlarged and thickened after long-continued inflammation of the synovial membrane," &c. &c.

M. Bouillaud had previously alluded to the influence of increased energetic action on the hypertrophic enlargement of muscular tissue, and had quoted with approbation the observation of Corvisart, that "exercise in the case of the external muscles, exercise and irritation in that of the heart, are the principal causes of the more active nutrition of these organs, and consequently of their unusual development."

explained in the present, as well as in the preceding article—is seldom, if ever, truly *curable.* Its effects may be mitigated, and its increase may be much retarded by wise treatment; but there is no satisfactory evidence, he thinks, to shew that a really hypertrophied heart has ever been reduced to its natural dimensions. It would be important if we could determine this point with accuracy; but as yet we have not sufficient data for the purpose. Our own experience would lead us to adopt the same views with our author. But, whatever opinion may be entertained on the subject, no experienced physician will hesitate to give his ready assent to the wisdom of the remark that, in the treatment of actual Hypertrophy, we must be on our guard not to take the mere force of the pulse in determining the amount of blood to be taken by venæsection, when this is deemed requisite. The object is not to *cure,* but to *relieve,* the disease. From four to eight ounces will be amply enough. The operation may require to be repeated at intervals of some weeks or months, its effects on the general system, as well as on the aggravated actions of the heart, being all the while most attentively watched. But often, very often, the local detraction of blood from the cardiac region is decidedly to be preferred to bleeding from the arm. Dr. Latham very wisely recommends that the application of a few leeches should invariably precede the use of the lancet; it is often astonishing how much relief may thus be promptly given.

When Hypertrophy is associated with Anæmia, the case is always a very serious and unmanageable one. Such a complication may have been induced by the injudicious depletion of blood. "Beware then," says our judicious author, with marked emphasis, "in the management of hypertrophy of the heart, beware, above all things, of bleeding your patients into paleness and poverty of blood."

The following passage, descriptive of the most frequent of all the complications of Hypertrophy, viz., with Valvular Disease, is so replete with interest in a physiological, as well as in a practical, point of view, that we shall give it entire.

"Here the diagnostic signs of both remain, so that there can never be the least doubt of the existence of either. The impulse within the chest, constantly augmented in degree and in extent, denotes the hypertrophy. The endocardial murmur, constantly present, denotes the injury of the valves.

"But, while the coincidence of the two is thus far without prejudice to the diagnostic signs of either, yet looking further to the actions of the heart itself, to the movements of the blood within the arteries, and to the deeper and more vital derangements, which naturally belong to each when they exist separately, we clearly discern the effects of the one wonderfully merged and lost in the effects of the other, now that they exist in combination; we see the effects of valvular injury merged and lost in those of hypertrophy.

"To valvular injury naturally belong an irregular contraction of the ventricle and an irregular pulse, and obstructions and delays to the course of blood through the arteries in various measures according to its degree. To hypertrophy naturally belong an excessive force of contraction in the ventricle and an excessive impulse communicated to the current of blood in the arteries. And this force and impulse are naturally opposed to all that is irregular and eccentric in the action of the heart and arteries, and even counteractive of it, when it would otherwise arise. Thus, they are counteractive of their irregular action, when it otherwise would arise from valvular injury.

"Moreover this coincidence of hypertrophy of the left ventricle and of an injured valve exhibits the most beautiful example, in the whole range of pathology, of the checking, redressing, and compensating powers which nature possesses and uses in furtherance of the great ends of mitigating distress and of protracting life, when some important structure is damaged beyond the possibility of reparation.

"In this coincidence there is nothing of accident; all is of design. The important structure, damaged beyond the possibility of reparation, is the valve. The unsoundness of the valve comes first, and then produces the hypertrophy, and produces with it the redress of its own injuries. While the valvular unsoundness is yet small, and still when it has become greater, and even still when it has become very great, the heart is often found from first to last maintaining its rhythm and the pulse its regularity. And no wonder. For it is accompanied at every stage of its increase by a proportionally increasing power of the ventricle.

"A loud systolic endocardial murmur and an excessive impulse of the heart and a larger space of præcordial dulness than natural, these are the sure and authentic signs of an injured valve and hypertrophy of the left ventricle. Yet often and often are these found to co-exist, when the order and sequence of the heart's contractions and the beats of the pulse are perfectly regular and rhythmical. And further, with this certain evidence of an injured valve and of hypertrophy of the left ventricle, not only will the heart and the pulse beat regularly, but the blood will continue to be distributed freely and equably throughout the body. Often the complexion is still healthy, the lips florid and the body well nourished.

"Here it is the hypertrophy, which is the safety of the patient and enables life to go on as it does. Take away the hypertrophy and leave the injured valve, and the patient would be in a far worse state than he now is; worse with half his disease than he now is with the whole of it. The pulse would begin to flutter, the complexion would become dusky and the lips blue, and the surface of the body mottled and patched in consequence of the blood being here and there unequally distributed or partially detained. The ventricle reduced to its common bulk would want the power needed to impel the blood steadily onwards against an extraordinary obstacle." P. 297.

Here then we have a striking illustration of the *vis medicatrix* (or *conservatrix) Naturæ.* The obstruction to the free exit of the blood from the heart is fixedly mechanical and altogether irremoveable. The only way, therefore, to meet the difficulty is to increase the force of the pump. This is what Nature does. The remedy, it must be confessed, is the induction of a new disease: but this is rendered inevitable by the pre-existing one.

In the treatment of Hypertrophy of the Heart, we are always to bear in mind that this morbid change seldom continues long without being accompanied with disease—usually of an inflammatory or of a hæmorrhagic character—in some other organ or part of the system. The Lungs, the Liver, the Kidneys, and the Brain are, each and all of them, exceedingly apt to become the seat of some irregular and morbid action. Perhaps, however, the general Arterial system itself is what most frequently and most severely suffers, and in which the most constant marks of structural change are discoverable upon dissection.

Dr. Latham has very wisely directed the attention of his readers to the very important fact, in the history of chronic cardiac disease, that not only the dynamic forces of the circulation, but that also the quali ies of the circulating fluid itself become, in course of time, almost inevitab.y injured.

Hence, unquestionably, the tendency to congestions, dropsical effusions, and hæmorrhages is vastly increased beyond what we may reasonably suppose would be the case, if the blood itself remained in a completely healthy condition. To omit, therefore, from our consideration the most important element in the production and aggravation of so many maladies—we mean that of *humoral* change—is at variance alike with the rational physiology of disease and with its scientific and truly successful treatment. The author says:—

"The general mind of the profession is just now all alive in quest of the elements of disease in the blood. A good deal is in a hopeful way of investigation, and some little is already made out. But let us beware of the common fault of physicians in all ages, and not make too much of our new knowledge and call upon it prematurely to explain everything. Thus much, however, we cannot help seeing plainly enough, that the opposite states of plethora and anæmia have a vast pathological import both in themselves and in relation to all diseases, come from what source they may. They have it unquestionably, and they display it in relation to those secondary diseases which spring from an unsound heart.

"Plethora belongs essentially to the blood, and results from one of its elements, the globules, being in excess. Now think of what plethora is in its effects; how it modifies the functions of health, how it directly conduces to certain kinds of diseases, and how it stamps a peculiar character upon all.

"Think of great habitual force of the heart's action and great habitual fulness of the pulse; of blood carrying with it its visible colour of blood much further into the capillaries than natural; of rapid digestion and rapid nutrition, great consciousness of strength and vitality, and great muscular development. Such is the health of the plethoric.

"Think of frequent vertigo and ringing in the ears, and frequent drowsiness; of spontaneous congestions and spontaneous hæmorrhages and feverish heat on slight provocations. Such are the proper ailments of the plethoric.

"Again, anæmia belongs essentially to the blood, and results from one of its elements, the globules, being in defect. Consider what anæmia is in its effects; how it, like plethora, modifies the functions of health but in a different way; how it too conduces to certain kinds of diseases, and how it stamps a peculiar character upon all.

"Habitual feebleness and frequency and occasional irregularity of the heart's action, habitual smallness of the pulse, the blood failing to give its colour to the skin and to the visible portions of the mucous surface; slow and painful digestion, defective nutrition, cold extremities, nervous depression, mental irresolution, such are the ingredients which go to make up the health of the anæmic at best.

"Throbbing in the head, and vertigo and ringing in the ears as frequent as in the plethoric, and pain more frequent and more acute; also spontaneous hæmorrhage as in the plethoric, but now taking the shape of purpureous spots and blotches; and œdema of the ankles and feet, these are the proper ailments of the anæmic.

"Then every accidental form of injury and disease putting on the character of weakness; inflammation itself failing to accomplish the proper work of inflammation for want of power, and not bearing the remedies of inflammation, yet still continuing pertinaceously, and often refusing to be cured; such is the character they have in the anæmic.

"Now anæmia, bare anæmia, is a thing formidable enough in itself. Without disease or injury of any solid structure whatever, the essential disorder of the blood alone may kill. It may give occasion to passive effusions, and to passive hæmorrhages, and to passive inflammation, which bring on death.

"As in plethora, so in anæmia, each unsoundness of the heart becomes conditionally a worse disease, but worse in a different way. As in plethora so in anæmia, each unsoundness tends more rapidly to its evil consequences, but those consequences are apt to emerge in a different manner. Passive effusions, hæmorrhages, and inflammations are rather wont to appear everywhere than in certain parts. Death seldom now arrives by oppression of the brain or the lungs singly, but oftener by oppression of many organs simultaneously." P. 312.

So much for the important subject of Hypertrophy—simple and complex—of the Heart. We pass on to notice the opposite morbid condition, viz. *Atrophy*.

We find the following few remarks on its (supposed) causes in M. Bouillaud's work.

"The causes of this—by no means imaginary, as has been alleged by certain writers, and indeed tolerably frequent—disease are either local or general, direct or indirect, mechanical or vital. Among others may be enumerated, 1, long-continued compression of the heart from an effused fluid within the pericardium, or from some other mechanical cause; 2, a local impediment to the circulation on which its nutrition depends, by contraction or obliteration of the coronary arteries; 3, various hygienic and diseased conditions, the effect of which is to induce a state of general wasting or marasmus: the majority of the cases of Atrophy, which we have met with, have occurred in persons affected with some chronic, tuberculous, or cancerous, &c. disorganisation of the chief viscera, or in whom inflammatory disease had proved fatal after two or three months' persistence; and 4, an anæmic state of the system."

The form of Atrophy of the Heart, which is by far the most frequently met with, is that where the muscular tissue of one or of both of the ventricles becomes attenuated, and (as is usually the case) softened at the same time. We briefly noticed, in our last article, the effects of active Carditis in giving rise to actual suppuration in the ventricular walls, and we shall again have occasion to revert to M. Bouillaud's description of this very rare lesion. At present, however, we have to do with a very different form of the disease; that, namely, where there has been a wasting or disorganising absorption of the muscular fibres, so that the fleshy parietes of the heart often become so thin and softened as readily to give way under the pressure of the finger. In many cases, it is impossible to trace this most serious organic lesion to any probable cause. We have already alluded to instances where men, in seeming good health at the time, have been suddenly stricken with death, and in whose bodies the walls of one of the ventricles have been found reduced to the thickness of a line or two. In other cases, we are not without data for forming a reasonable conjecture, at least, upon the subject. For example, Softening of the substance of the Heart has been observed to occur in certain asthenic states of the system, in which there is a loss of tone or power in the entire muscular system; this atony originating from a diffluent, or otherwise depraved, condition of the blood itself. One of the most important varieties of this morbid lesion is that which so often occurs in Typhus and other low fevers.

"When in fevers the skin becomes dusky, and the impulse of the heart fails and fails, until it can be *felt* no more; and the systolic sound of the heart fails and fails until it can be *heard* no more; and death follows, and after death the heart is found to yield and fall in pieces under pressure of the fingers; then surely we cannot be wrong in ascribing to the softened heart a large share in procuring the fatal result.

"Again in fevers when the skin is dusky, and the impulse and systolic sound of the heart both fail, and death is imminent and threatening, and yet under the seasonable use of wine and stimulants the skin brightens and the heart is again felt and heard, and with its returning impulse and sound all inauspicious symptoms are gradually cleared up and recovery is finally complete, then surely we cannot be wrong in believing, first that the heart had been softened, and had afterwards recovered its natural texture and power, and secondly, that this recovery of its natural texture and power was mainly instrumental in saving life.

"This softening of the heart in fevers is no new fact. But the knowledge of the precise auscultatory signs which denote its softening, and of the precise auscultatory signs which denote its recovery, this indeed, is new knowledge, and we owe it to Dr. Stokes of Dublin. And further the detection, in these same auscultatory signs of one precise and plain *indication* to guide us in a most difficult point of medical practice, viz, the administration of stimulants in fever, this too is new, and this too we owe to the same sagacious physician. Whoever discovers a single new indication of treatment, which shall prove just and true and comprehensive, does a better service to mankind than if he found out twenty new remedies." P. 264.

A similar condition of the muscular substance of the heart has been observed in Scurvy, Chlorosis, and such-like diseases.

Dr. Latham is of opinion—contrary to that of several writers on the subject—that the softening of the heart, induced by Fever, Scurvy, or Anæmia, very rarely terminates in a permanent lesion of that organ, after the fever, &c. has long ceased to exist. The cure of the producing disease appears to be generally sufficient to effect a cure of the cardiac affection.

The softening, that occurs independent of all constitutional cachexy, is of a much more serious character; it is, indeed, one of the most dangerous of all cardiac diseases. It generally occurs in persons advanced in years, or prematurely old from excesses or irregularities in living; and, before it is recognised, there is almost always some other visceral disease, such as enlargement of the Liver, granulation of the Kidney, &c., or perhaps extensive arterial degeneration, associated with greater or less derangement of the nervous system. Besides, the heart in such cases is not merely softened; it is also very generally either hypertrophied or it is attenuated; and one or both of the ventricles are very usually dilated at the same time.

Before proceeding to notice any other diseases or morbid conditions of the heart, let us here dwell, for a few minutes, upon some of the most frequent and serious lesions that are apt to be induced in several of the viscera by organic changes in the central organ of the circulation.

As might be reasonably conjectured, the organs which suffer first and most severely in almost all cardiac diseases are those of respiration; in other words, the Lungs are the most frequent seat of the congestions and effusions, hæmorrhages and inflammations, which are the results and concomitants of heart-disease. The converse, too, of this proposition is equally true; for never are the cardiac symptoms so promptly and so distressingly aggravated as when pulmonic congestion or inflammation supervenes; and, as cases of this complication of evils are of very frequent occurrence, it behoves the medical man to be thoroughly acquainted with the phenomena which declare its impending or actual existence. The truth of the following graphic description will be recognized by all experienced practitioners.

"A man has hypertrophy of the heart in a moderate degree with some small amount of valvular injury or with none at all. Hitherto he has been tolerably free from painful palpitation and dyspnœa except under excitement or extraordinary exertion. But suddenly he is found gasping and struggling for breath and expecting instant dissolution. What is this; and what is to be done? Truly one might be excused for thinking of angina pectoris or some spasm of the heart, and flying to ammonia and æther and opium for relief. But putting my ear upon the chest I have found a small crepitation diffused through the half of one lung, or in the half of one lung I have been unable to catch any audible murmur whatever, either natural or morbid. A single cupping upon the chest, just opposite the portion of the lung that labours, has swept away the crepitation, or has removed the dulness and brought back the respiratory murmur; and the patient has been restored in a day or two to his ordinary state of comfort. Here in one instance there has been sudden and extensive effusion into the extreme bronchial ramifications or vesicular structure of the lung, and in another there has been sudden and extensive congestion.

"Such cases as these are very striking. All that belongs to the disease and all that belongs to the remedy is so clear, so marked and unequivocal; pulmonary congestion and effusion suddenly coming on and life brought into instant peril; and then, with the use of the proper remedy, congestion and effusion suddenly gone and life restored to safety." P. 321.

The direct anatomical connexion between the Circulatory and Respiratory organs sufficiently accounts for their close and very immediate sympathy in a state of disease. Nature has evidently designed that the lungs should, when occasion requires, act as a channel of relief to the heart, when it is oppressed with blood which it cannot easily discharge. Pulmonary Congestion is an almost invariable accompaniment of advanced cardiac disease; and the profuse bronchial secretion, and occasionally also the expectoration of blood, are only means by which the system seeks for relief. It is therefore by duly regarding these and such-like symptoms as processes set up by Nature to relieve first the congested state of the lungs, and secondarily the oppressed condition of the heart, that the physician will be best enabled to conduct the management of such a case. A *cure* is not to be thought of; but much may be done by judicious treatment to mitigate the many distresses that are inevitably present. The grand object should be to watch how Nature herself is seeking relief to herself, and to assist her in the effort; at one time by cautiously unloading the gorged state of the blood-vessels, by means either of a few leeches or of the cupping-glasses applied over the seat of the pulmonic congestion; at another, by attenuating the bronchial secretion and promoting its more abundant and easy discharge.

"Thus for months and months together and even for years, we may keep people alive and give them incalculable comfort by aiding the lungs in the office of relief, which they are striving to perform, to an unsound heart. This is done by drawing blood from, or by producing vesication or counter-irritation on, the walls of the chest, just when and where and to the extent that may be required. We should endeavour to make out from time to time, by careful auscultation, what parts of the lungs chiefly labour, where they chiefly crepitate or where they are becoming dull, and to the surface of the chest immediately opposite those parts apply our remedies. Thus by taking a few ounces of blood by cupping or leeches, or by applying a blister or a mustard poultice on the *right* spot and at the *right* time, we shall often obtain a degree of relief for our patient, which nothing but experience could lead us to expect. And we shall obtain the like relief in the same case again and again, always provided we take the same care to choose the right spot and the right time in the application of our remedies to the walls of the chest. And what is the secret of our success? The secret (I believe) is this. We are aiding Nature in the very channels through which she is seeking to obtain succour for herself. Therefore our remedies are the more effectual." P. 324.

The influence of *digitalis* in many such cases, and more especially when the action of the heart is unusually violent, is often of the most marked benefit. We must be cautious however, in the use of this potent medicine, not to administer it in such doses, or continue its administration so long, as to bring down the action of the hypertrophied heart to, or below, the standard of health.

We need scarcely say that the pulmonic disorders coincident with an hypertrophic and over-active state of the heart are, on the whole, much more manageable than those, which accompany an attenuated and perhaps softened condition of this organ and a feeble action of the general circulation. It may still be necessary to relieve the congested state of the pulmonary vessels by the use of leeches or of the cupping instruments applied over the seat of the mischief; but the relief thus obtained is neither so decided nor so abiding as in the former case. While we mitigate the local malady by such means, it *may be* necessary, at the very same time, to sustain the flagging powers of the heart itself by cordials and gentle stimulants.

The Brain is probably, after the lungs, the organ which most frequently suffers from organic disease of the heart. When the right ventricle is dilated and weakened, the patient will be exposed to the risk of cerebral congestion, and consequently of an apoplectic or paralytic seizure. On the other hand, if the left ventricle be enlarged and thickened, the opposite condition, that of high cerebral excitement, is apt to ensue. "The head is racked with continual pain; there is little or no sleep; the patient becomes delirious, then maniacal, then convulsed, and he at last sinks from exhaustion of his nervous system."

Among the other secondary disorders attendant upon, and indeed induced by, cardiac disease, especially when the right cavities of the heart are chiefly affected, enlargement, congestion and various disorganizing structural changes of the Liver and Kidneys deserve the attentive consideration of the physician.* The dropsy, that so generally complicates the

---

* M. Bouillaud has the following observations:—"Among the influences that are apt to retard the pulsations of the heart, must be enumerated all gloomy, sad, and depressing emotions of the mind. The presence of the elements of the bile in the blood occasions a remarkable retardation of the pulse. In almost every case of apyretic Icterus, we shall find that such is the case. This curious phenomenon, which has hitherto escaped the attention of physicians (?), has been observed by me in nearly 200 instances. In certain persons of the bilious or melancholic temperament, the pulse has been remarked to be usually slower than in other individuals. Corvisart tells us that Napoleon's pulse seldom exceeded 60 in the minute. I have noticed the same thing in other persons of the same temperament, and in whom the mental energy was great."

latter stages of heart-disease, may, in a very great many cases, be traced to the disordered condition of these two great viscera as its more immediately exciting cause. Hence the obvious importance of assiduously attending to the state of the biliary and urinary secretions in the treatment of all organic affections of the heart, and particularly in those which are seated in its right cavities, and in which there is consequently a greater or less obstruction to the free return of the venous blood. The engorgement of the Liver that is thus induced is known to every medical practitioner; and it seems highly probable that the albuminous condition of the urine, which is not unfrequently observed to be present in cases of cardiac disease, is (often at least) attributable to a congested state of the Kidneys, arising in the manner alluded to. But we cannot pursue this subject at present; and must pass on to other matters.

On the very important subject of Dropsy in connexion with cardiac disease, Dr. Latham remarks, with his accustomed analytic skill—

"In every case of unsound heart the first appearance of the least dropsical symptom marks an eventful period: it marks the period when a new law is beginning to take effect in the circulation and to gain a mastery over the law of health. The law of health, of which the sound heart is the prime agent, retains the blood within the blood-vessels, or dispenses it only for the needs and uses of health. The new law, of which the unsound heart is still the prime agent, suffers or forces the blood or some of its constituents to escape and to form accumulations of serum out of the courses of health.

"A little œdema of the ancles or a little œdema of the face is a sufficient notice of the first yielding of the blood-vessels to this new law, which is no other than a mechanical necessity against which they can no longer hold out. It is the earliest beginning of serous effusion, which may go on increasing until it has pervaded the entire cellular structure and filled every serous cavity of the body.

"All this calls at once for medical treatment. But what is to be done? The circulation *must* have the relief it seeks somehow. Disburden itself it *must*. There is a physical necessity in the case from which we cannot set it free. Or if we could, it must be by interfering remedially with the actuating cause, *i. e.* by curing the unsound heart. But this is impossible. What then can be done?

"This can be done and this only. Seeing what nature is doing and must do, we can only go along with her and seek to aid her in accomplishing her own purposes through other and less hazardous channels. Nature is seeking relief by directly evacuating the blood-vessels of their contents. We must try to gain for her the same relief by augmenting natural secretions and so evacuating the blood-vessels through natural channels. The kidneys and the intestinal canal and its subservient viscera are the most eligible for the purpose." P. 346.

We need scarcely say that the success of our treatment will depend very materially upon the circumstance, whether there be coincident disease in some of the other internal viscera—the lungs, the liver, or kidneys—besides the heart itself, and whether there be any cachectic condition of the blood itself or not, at the same time.

*Angina Pectoris.*—To the consideration of this frightful and mysterious malady, Dr. Latham devotes his two last Lectures. Its essential characters are, it is well known, an agonizing pain in the region of the heart or immediately under the sternum, accompanied with a sense of imminent dissolution. These are its essential and pathognomonic symptoms. They are often attended with others, but not necessarily so. The most frequent of these is the extension of the pain to one or both arms, most frequently to the left, and stopping at the elbow. Dr. Copland's definition of the disease is, on the whole, exceedingly good :—

> " Acute constrictory pain at the lower part of the sternum, inclining to the left side, and extending to the arm, accompanied with great anxiety, difficulty of breathing, tendency to syncope, and feeling of approaching dissolution."—*Dictionary,* Vol. I., p. 62.

The agony is generally sudden in its invasion, and often equally sudden in its cessation. It is now universally admitted, that the disease is of a spasmodic or spasmodico-neuralgic nature, and that it is not necessarily associated with any one organic alteration of the heart in particular. It has been found, " where there has been ossification or obstruction of the coronary arteries, where there has been dilatation of the aorta, where there has been valvular unsoundness, or hypertrophy or atrophy, or softening or conversion of the heart's muscular substance into fat. It has been coincident with one and one only of these forms of disease or disorganisation, or with two or more of them in combination. And it has existed where no form of disease or disorganisation whatever has been found either in the heart or in the blood-vessels nearest to it."*

The three cases, whose histories are detailed by Dr. Latham, are, each and all of them, very interesting.

The *first* occurred in a gentleman 50 years of age, who had felt himself in perfect health until within a fortnight of his first applying for medical advice. Indeed, in the preceding Summer, he had made a walking tour through Switzerland ; and, after returning home hearty and well, had enjoyed the sport of shooting during the Autumn. It was while walking up the hill towards Hampstead, one day in the beginning of January, that he first experienced an attack of angina. During the next fortnight, the paroxysms increased in severity and frequency. At first they occurred every two or three days, then daily, and now, (when he consulted Dr. Latham,) several times a day; at first with, and now without, a cause. The only irregularity discoverable, upon auscultation of the chest, was an " exceeding feebleness of the heart's impulse." Next morning, he had a paroxysm more severe than any he had yet experienced ; but when Dr. L. saw him, he looked to be quite well. In the afternoon, however, of the same day, he was seized with another paroxysm and died at once.—*Dissection.*—" The heart was of its natural size, its cavities of their natural capacity, its walls of their natural thickness, and its internal lining and valves bearing no marks of disease. Its muscular substance was more loose of texture than natural, but not softened in an extreme degree, and both its coronary arteries were entirely converted into calcareous tubes as far as they could be traced. The aorta throughout the chest and the abdomen did not present the smallest space free from disease. In some parts calcareous matter was deposited between its coats, in others cartilaginous, and in others a matter between cartilage and bone. This disease, besides

---

* Perhaps attenuation and softening of the muscular parietes of the cardiac ventricles are the lesions that have been found more frequently than any other in fatal cases of Angina Pectoris.—*Rev.*

destroying the elasticity of the aorta at every part, had greatly narrowed its calibre at a small space of its descending portion within the chest, and so produced some real impediment to the passage of blood. We did not examine the state of other bloodvessels. Other viscera of the chest and abdomen were healthy, except that there was a close and complete adhesion of the pleura of the right lung to the ribs without the least apparent detriment to the lung itself."

The most remarkable feature in this case was the shortness of the interval between the first paroxysm and the one which proved so suddenly fatal; and this too in a man who had previously appeared, and felt himself to be, in perfect health.

In the *second* case, which occurred in a robust man 64 years of age, the heart was found on auscultation to be "beating with a perfect rhythm, and neither with excess nor defect of impulse. The sounds were natural but loudly intonated, and conveyed over the front of the chest far beyond the præcordial region." The respiratory murmur was normal everywhere. The patient had believed and felt himself to be in perfect health, when he was suddenly, and without any apparent cause, seized with a paroxysm of severe pain, passing from the upper part of the sternum through the chest to the back, and down both his arms to the tips of his fingers. The second attack occurred four days subsequently. From that time, the paroxysms were numerous by day and night. The change from the erect to the recumbent posture always produced them. Their duration was from five to ten minutes. Dr. Latham saw him on the ninth day (from the first seizure), and on the following day, after having had eight or nine paroxysms, he died. *Dissection.*—"The pericardium was found to contain two ounces of clear serum, and upon its surface covering the heart it presented a few small white spots. The heart itself had an appearance of general enlargement. Its internal lining and all its valves were healthy, except that the processes of the mitral valve might be thought a little thickened, but not so as to hinder the circulation. The coronary arteries too were quite healthy. But the muscular substance of both ventricles was so soft as to be pierced through with the slightest pressure of the finger. The aorta was entirely free from all morbid deposits. Both pleuræ were free from adhesions, and contained no fluid in their cavities. Both lungs were entirely healthy, and so were all the abdominal viscera." In this case, too, the fatal rapidity of the disease is the circumstance most worthy of notice.

The *last* instance, related by our author, is that of the late most estimable and deeply-regretted Dr. Arnold of Rugby, the history of whose last illness is so feelingly recorded in his "Life and Correspondence." In his case, (he was 47 years of age,) up to a very few hours before his death, both body and mind seemed equally to give proof and promise of health. He still took his accustomed pleasure and refreshment in strenuous exercise. He retired to rest at midnight on the 11th of June, 1842, feeling and believing himself to be in perfect health. About five o'clock next morning, he was seized with a very severe pain seated at the upper part of the chest towards the left side, and extending down the left arm. It recurred at intervals. When Dr. Bucknill saw him at seven o'clock, the breathing was not disturbed; but the pulse was exceedingly feeble, and scarcely to be felt at the wrist. Brandy and water was given, and the pulse became natural. The pain returned, and again the pulse became very weak. The paroxysm was short, and he soon recovered himself. While Dr. B. was mixing an anodyne draught, he heard a rattling in the throat, and a convulsive struggle. He turned round and saw that his patient was in the agony of death. The eyes were fixed, and the teeth set. The breathing was very laborious, the chest heaved, and there was a severe struggle over the upper part of the body. The pulse was quite imperceptible, and, after deep breathings at a few prolonged intervals, all was over. Dr. A. died in little more than half-an-hour after Dr. Bucknill's arrival.

*Dissection.*—Both lungs posteriorly were gorged with blood and serum, and about eight ounces of bloody serum were found in each pleural cavity. "The pericardium was healthy. It contained about an ounce of serum of a straw-colour. The heart was rather large. The external surface was healthy. It was very flaccid and flat in its appearance. It contained but little blood, and that was fluid. There were no coagula of any kind in it. All the valves were quite healthy, and so was the lining membrane throughout. The orifices of all the great vessels were quite natural. The muscular structure of the heart in every part was remarkably thin, soft and loose in its texture. The walls of the right ventricle were especially thin, in some parts not much thicker than the aorta, and very loose and flabby in their texture. Its cavity was large. The walls of the left ventricle too were much thinner and softer than natural. And the muscular fibres of the heart generally were pale and brown. The aorta was of a brown red colour throughout its internal surface, probably from putrefaction. A few slight atheromatous deposits were observed in the descending thoracic aorta. The pulmonary artery was of the same brown-red colour with the aorta. There was but one coronary artery, and, considering the size of the heart, it appeared to be of small dimensions. It with some difficulty admitted a small director. It was slit open to the extent of nearly three inches. Its internal surface was red but healthy, with the exception of a slight atheromatous deposit situate about an inch from the orifice of the artery. This however did not appear to diminish its cavity."

In the two first cases, we counted the duration of the disease by days; in this we must count it by hours. Not three hours elapsed between the earliest intimation that anything was wrong with the functions of the heart, and the moment when "the spirit returned unto God who gave it." Very rarely does the stroke follow so quickly upon the first not-to-be-mistaken announcement of its imminence. In most cases of Angina Pectoris, the life of the patient is prolonged for months, and even years; and, very generally, there is complex organic lesion of the heart and great blood-vessels present. The three cases, therefore, which we have now briefly recorded, are especially interesting from their fatal rapidity. It is unnecessary to analyse their peculiarities, or the differences that may be traced either between themselves, or between them and other cases on record. Suffice it to say that they cannot be very satisfactorily quoted to prove the organic or mechanical theory of the cause of Angina. The truth seems to be that, although a structural change of the heart is unquestionably present in a very large majority of cases, the lesion is not the immediate or direct, but only the predisposing, cause of the disease; the essential one being a *spasm* or cramp of the muscular substance of the heart.*

"From what we know of pain and spasm, and the things causing and pertaining to them, in other parts of the body, we might be prepared for the same sort of things causing, and pertaining to, them when they belong to the heart. Thus we might expect to find Angina pectoris incident to any form of organic disease of the heart, but constant to none. And such is the fact. We might expect to find angina pectoris where there was no organic disease of the heart itself, but such organic disease elsewhere as might injuriously interfere with the functions of the heart. And such is the fact. And finally we might expect to find angina pectoris, where there was no detectible organic disease either of the heart or of other parts, but where itself (namely spasm) constituted the whole disease; a disease purely vital, a disease of feeling and function alone, operating by and through sound structure, it may be fatally, always perilously. And such is the fact.†" P. 387.

The following most judicious remarks on the management of patients, who are subject to the formidable disease of *angina pectoris,* will not fail to be read with interest by every one engaged in practice.

"The paroxysm is often put off and its severity mitigated and life prolonged by no means more surely than by keeping the vascular system in a just balance between fulness and emptiness, between rich blood and poor blood. In some constitutions, very happily born, the balance maintains itself; and then there is no need of interference on our part. In the majority it is not exact, yet exact

---

* This seems to be the meaning of the word "distensionem" used by Heberden in his excellent description (the first that was given), to designate the probable proximate cause of the disease. Possibly, also, the idea of the cavities of the heart being so *distended* with blood as to be unable to contract and propel it forwards is implied in the teim. The following is the passage alluded to:—

"Angina pectoris, as well as I have yet understood its nature, seems to pertain to *distension*, and not to inflammation:

For, first, it comes suddenly and goes suddenly.
Next, it has long and complete intermissions.
Then, wine and spirituous drinks and opium afford considerable relief.
Then, it is aggravated by agitation of the mind.
Then, for many years, it distresses a patient without other injury of the health.
Then, at first it is not excited by carriage or horse exercise, as is usually the case when there is scirrhus (induration or organic disease) or inflammation.
Then, the pulse is not accelerated even in the paroxysm.
Lastly, in some the attack occurs after the first sleep; a circumstance which is common in diseases arising from *distension*."

After alluding to the absence upon dissection of all organic lesions in the heart and great vessels, save only a few small bony specks in the aorta, of one of his patients who died from this disease, Dr. H. remarks that, in several cases, he had observed that the blood retained its fluidity after death, so that it continued to ooze from a wound in a vein, as long as the body remained unburied.—*Commentarii de Morborum Historiâ et Curatione.*

† "The fact is not within my own experience; but I must admit what is credibly reported."

enough for the ordinary purposes of health, but not enough when there is some grave infirmity to be palliated and made tolerable elsewhere. A small habitual deviation on this side or that is readily felt and resented by the heart, when it has undergone some form of unsoundness rendering it obnoxious to spasm.

"Thus there have been cases in which my treatment of angina pectoris, in the intervals of the paroxysms, has chiefly turned upon reducing the nutritious and stimulant quality of the patient's diet, abridging his animal food, and denying him wine and fermented drinks altogether. There was one case, and only one, in which I was driven to draw blood even more than once from the arm; an unusual and a hard necessity! There have been more cases, on the other hand, in which the general habit of the patient has made me fearful of withdrawing support, and experience has shown me the need of supplying a well-regulated amount of stimulus in the shape of wine daily. The administration of steel in the intervals of the paroxysms has (I have convinced myself) in some instances been instrumental to their postponement.

"Truly a volume might be spoken upon the subject, if one were to enter into the detail of all the indications, which the general vascular system may offer to the observant physician, for the employment of remedies as a safeguard, within all possible limits, against the attacks of this awful disease.

"And truly the same may be said of the nervous system, and how it notifies indications of treatment by its various states of disorder; and how it presents itself as an avenue for remedies, which may carry a salutary impression to the heart and withhold it for a time from falling into spasm. Loss of sleep, disturbed sleep, and painful irritation and troublesome wants, such as frequent micturition, may be among the bad habits of the patient, or they may be induced by his disease, or they may be aggravated by it. At all events they contribute to bring on the paroxysm more frequently and more severely. It is wonderful what a small quantity of opium, administered dexterously upon such indications, will sometimes do in keeping angina pectoris from advancing to a greater degree of suffering, or in bringing it back from a greater to a less." P. 407.

A dose of Paregoric and sulphuric Æther on going to bed, and again in the morning before rising, will often be found an excellent remedy in keeping the uneasy feelings about the heart at bay. The application of epispastics over the region of the heart, or the establishment of a perpetual irritation there by seton or otherwise, will in many cases be found to exert a decidedly beneficial effect in diminishing the frequent recurrence of the cardiac paroxysms. We need not say anything on the score of bodily exercise or of mental excitement. The poor invalid soon discovers how inevitably any indiscretion, either of mind or body, aggravates his distress. And surely it is nothing but the duty of a good physician to endeavour to direct his patient's thoughts to the habitual contemplation of those subjects, which will assuredly bring him more perfect tranquillity and a more settled evenness of feeling than all the resources of art or the unaided resolves of weak humanity can accomplish. Nothing but the deep and assured conviction that "all is well with him," can enable the sufferer to anticipate and meet with composure the fearful inward agony, which is never far distant, and which may overtake him in a moment, snapping the cord of life ere relief can be found, or the slightest preparation for death can be made. The thought is indeed a solemn one to all:—what *ought* it to be to one who may be suddenly

> "Cut off even in the blossoms of his sin,
> Unhousel'd, unappointed, unaneal'd,
> No reckoning made, but sent to his account
> With all his imperfections on his head?"

M. Bouillaud slurs over the subject of Angina Pectoris, as if it were a matter of little interest. Indeed, it may be remarked generally of his bulky work, that the reader will find but very little to engage his attention in the description of those diseases, which may be said to be rather vital than physical or material in their phenomena and effects. He is, as might be expected, most elaborate and diffuse in his account of the inflammatory affections of the heart. We shall briefly allude to his opinions on one or two points in the history of those diseases.

He considers that there are two primary kinds of Endocarditis—1, the actively *inflammatory*, such as is so often present in Rheumatism, and in severe Pneumonia and Pleuritis; and 2, the *septic* or putrid, as is found (so he says) to exist in Typhus, and Typhoid affections. "Doubtless," he adds, "there may be a *gangrenous* form of the disease also;" but he very wisely reminds us of our utter ignorance as yet of the subject.*

The description, which he gives of the necroscopic appearances of Endocarditis, is strangely unsatisfactory. For example, we are told that the redness of the endocardium is "owing not to any (at least appreciable) injection of its capillaries, but rather to a sort of dying or imbibition of

---

* M. Bouillaud appears to have his mind so entirely occupied with the connexion between acute Rheumatism and Carditis, that he does not seem to be at all aware that the inflammatory affections of the heart, and more especially of its inner membrane, have been of late shown to be, in many cases, associated with granular degeneration of the Kidneys. The recent very elaborate paper of Dr. Taylor, in the Medico-Chirurgical Transactions, (vide the No. of this Journal for April 1846,) has added much to our hitherto imperfect acquaintance with this important ætiological question.

Our readers are probably aware, that we have long advocated the doctrine that endocardial disease is (in all probability) often induced by vitiated conditions of the circulating fluid; and recent researches have certainly tended to confirm the truth of this opinion. The last occasion, on which we alluded to this subject, was in our review of the first volume of Dr. Latham's Lectures.

"Having thus, at very considerable length, examined the history of endocardial and pericardial inflammation, more especially in reference to its symptoms and its predisposing causes, the question naturally arises, what is the more immediate *cause* of the disease, and what peculiarity is there in Rheumatism for example, or in Scarlatina, that should be liable to induce an inflammatory affection of the Heart, rather than of any other organ? For our own part, we have long been inclined to believe that it is *to a state of the circulating fluids* that we must look for an explanation of this pathological phenomenon. Is it not reasonable, to say the least of it, that an altered condition of the blood may occasion an irritation of the lining membrane (more especially) of the heart? That such an alteration exists in the diseases which we have named—Rheumatism and Scarlet Fever—will be denied by none; and may not the same be said of the other states of the system, with which active cardiac disease has been observed to co-exist? The *ætiology* of Heart-affections is yet but very imperfectly made out. In the general run of cases, it will be difficult or utterly impracticable to ascertain the probable cause. In a good many, indeed, it may be found that the patient has, at some period or another, suffered from Rheumatism; but certainly not in the majority. Let us hope that the great attention that is now paid to humoral pathology will, ere long, throw some light upon this subject."—*Medico-Chirurgical Review for July*, 1845.

the affected surface." A few lines further on, we read that "the redness of the internal membrane of the sanguiferous system is sometimes of inflammatory origin, and at other times is the result of mere cadaveric imbibition. We must remain in doubt as to the real nature of this endocardial and vascular redness in some cases; where, the patients having exhibited during life the symptoms of an imperfectly-developed inflammation of the heart and blood-vessels, the *post-mortem* examination has been made at a time when incipient decomposition of the body has taken place. The uncertainty will be still greater, if putrefaction has fairly commenced, more especially when the patient has died from some typhoid affection, in which the dissolved state of the blood is more than usually apt to induce a sanguineous discolouration of most of the tissues. For my own part, I have been long in the habit of attributing it to the effects of inflammatory action, whenever it could not be reasonably ascribed to cadaveric imbibition."

In addition to increased redness, the Endocardium is frequently found to be swollen, thickened, and not so smooth as in health. These appearances are most conspicuous on the valves. Occasionally, this membrane is unusually friable, and is found to be much more easily detached than in the sound state. Now and then, its surface exhibits several erosions or points of ulcerative absorption.

In some cases, pseudo-membranous concretions, and, in others, genuine pus—either loose, or imbedded within a coagulum of blood—are met with in the cavities of the heart. The former, being possessed of great tenacity, become glued to the valves, and are interlaced with the *chordæ tendineæ*,* causing a more or less complete adhesion of their opposite surfaces, and eventually a contraction of the valvular opening. In other cases, the lymph is deposited in the form of granules or rounded vegetations on the loose edges of the valves, &c. In course of time, these granules become so dry and friable as to be readily crushed between the fingers. Of all the cardiac valves, the *bicuspid* is most frequently the seat of these deposits; those of the *aorta* are next in point of frequency. In a few instances, M. Bouillaud has met with a putrilaginous softening of the endocardium, accompanied with a gaseous infiltration of the muscular substance of the heart; phenomena which he has regarded as (probably) the results of a *gangrenous endocarditis.*

As in the case of inflammation of the internal membrane of the arteries and veins, so in Endocarditis, there is a marked tendency to the formation of *sanguineous concretions* or coagula within the cavities of the heart. These are thus described by our author.

---

* The following allusion by our author to the contraction of these *chordæ* is so truly French, that we cannot withhold it from the reader.

"For some years past, the profession has been much occupied with the subject of Deformities, for the relief of which the operation of Tenotomy has been employed with a varying degree of success. Among the lesions of the cardiac valves, and of their tendons, there are some which enter into the category of those to which we allude. But, alas! the affected parts are in some sort(!) sacred against the surgeon, and inaccessible to his instruments."

"These concretions, different from those which occur in the last moments of life, or immediately after death, are white, elastic, glutinous, adhering to the walls of the heart, just as pseudo-membranous concretions themselves, and, like these last, twisted around the tendons and the valvular laminæ. Analogous to the inflammatory crust of the blood, and imperfectly organised, they sometimes present to the eye, here and there, red points and lines which are in reality rudiments of vessels. They extend more or less deeply into the large vessels which proceed from the heart. Some of these concretions, deposited in the form of granules round the edge of the valves, become, as well as the pseudo-membranous granulations themselves, the origin of those vegetations of which we shall hereafter speak more fully."

After minutely describing the successive changes that take place in the cardiac cavities, and more especially in their orifices, from the time when the semifluid lymph is secreted during an attack of Endocarditis until the deposits acquire a horny or bony consistence, our author concludes with the following reflection:—

"The organic contraction of the orifices of the heart, so commonly the result of a lengthened endocarditis, is a new feature of resemblance between this phlegmasia and those inflammations which affect other hollow viscera. Who knows not, in fact, that the urethra, the neck of the bladder, the various excretory ducts of the tears, of the saliva, of the bile, and of the urine, and that different portions of the digestive tube, &c., may experience an organic contraction more or less considerable, in consequence of a chronic inflammation of their internal membrane, and that, as in the case of the heart, it is the narrowest points, viz. the orifices of the organs now mentioned, which are more especially the seat of the contraction?"

M. Bouillaud in general descants at much greater length upon the necroscopic effects of diseases than upon the phenomena or symptoms which they exhibit during life. Here is a specimen of his semeiological descriptions:

"The want of harmony or correspondence between the force of the cardiac and of the arterial pulse, in the advanced stage of endocarditis, is always a very unfavourable symptom. The heart may be labouring and thumping with violence against the ribs, while the pulse at the wrist is feeble and compressible. When such is the case, we may generally suspect the presence of fibrinous concretions in the cardiac cavities—and especially around the valvular orifices—which necessarily obstruct the free issue of the blood into the arterial trunks. The pallor or livid hue of the countenance, the jactitation of the limbs, the oppressive anxiety, the stiflings, and tendency to fainting, sufficiently attest the nature of the existing lesion. When the patient survives for some time, dropsy of the limbs and internal cavities is an almost invariable sequence."*

The following, not very satisfactory, tabular view of the discriminating symptoms between Endocarditis and Pericarditis is given:—

---

* Perhaps our readers may be interested to learn that Mirabeau died of carditis: his sufferings were so severe that he besought Cabanis, his physician, to put an end to them by large doses of opium. "Had Cabanis," remarks our author, "known and practised the method we adopt (bloodletting upon bloodletting, until the inflammation was arrested), he might have saved the life of his illustrious friend!"

| *Simple Pericarditis.* | *Simple Endocarditis.* |
|---|---|
| 1. If a considerable effusion exists, the hand is not sensible of the pulsations of the heart, and they are no longer visible. The sounds, in other respects normal, are remote from the ear. | 1. The pulsations of the heart are sensible to the hand and to the sight; the sounds of the heart are as near the ear as in the normal state, and are accompanied by a more or less rough blowing. |
| 2. If a moderate effusion exists so that the opposite layers of the pericardium may still be applied one against the other, a rubbing pericardiac sound is heard; a sound which in a manner is perceptible right under the ear of the observer, diffused, rough, a sort of *frou-frou*, which is like nothing but itself. | 2. The more or less rough blowing sound which is heard is deep-seated, and the ear readily discovers that it does not take place at the surface, but in the interior of the heart. It is usually audible at a greater distance than the pericardiac rubbing sound, and it is more circumscribed. |
| 3. This sound may sometimes be made to disappear by changing the position of the patient, in such a manner that the effused fluid becomes interposed between the layers which glide roughly one against the other. | 3. A change in the position of the patient never causes the sound in question to disappear. |
| 4. The pericardiac effusion occasions the dulness upon percussion to be more extended than in health; also a considerable prominence of the precordial region. | 4. The encreased extent of the dulness of the precordial region is inconsiderable, and, when any degree of prominence exists, it is but very slight. |

The attempts to distinguish the exact seat of organic cardiac disease have not, it is well known, been attended with very satisfactory success. Seldom have two authors agreed in their observations upon this point, which is fortunately more curious than of practical moment. M. Bouillaud, indeed, lays claim to extraordinary skill in the diagnosis of this class of maladies; but we are by no means inclined to pin our faith to all his assertions.

"The isochronism of the blowing, sawing, or rasping sound with the systole or with the diastole of the ventricles is not, *by itself*, of so much value as has been alleged by some superficial observers who have, under the vague term 'insufficiency of the valves,' confounded several valvular lesions, that are obviously distinct from each other. Let it not, however, be forgotten, that, in the majority of cases of constriction of one orifice, the abnormal sound is audible, both during the heart's systole and its diastole; but it is important to know that the regular click-clack of the valves, corresponding with the contracted orifice, is extinct or nearly so, while the click-clack of the sound valves continues to be heard. It is quite true that, when the abnormal murmur exists both during the systole and during the diastole, it masks, nay, sometimes entirely conceals, the sound of the valves which are healthy. By removing however the ear from the point where the abnormal murmur is most intense, we may by degrees distinguish the sound of the healthy valves. If, for example, there be contraction of the left auriculo-ventricular valve with loud abnormal murmur, audible alike during the systole and the diastole, the ear, applied immediately over or very near to the seat of the sound, will probably not succeed in distinguishing the *clacking* through the morbid murmur with which it coincides. But, if the ear be applied to the middle or upper part of the sternum, or in the subclavicular region, the auscultator will generally hear, more or less distinctly, the sound in question—which, as is well known, is produced by the *redressement* of the aortic valves during the ventricular diastole. We cannot too urgently recommend physicians, in their use of auscultation in cardiac diseases, to attend to the sounds as heard at different points of the chest. Without this precaution, it would have been a matter of great difficulty for us to have carried our diagnosis of various valvular lesions to that degree of precision and exactitude which may be now attained."

When the first of the two cardiac sounds is chiefly affected in the way of irregularity or other abnormal modification, we may suspect the lesion to be seated in the aortic valves; whereas, when it is the second which is so affected, it is probably in the auriculo-ventricular orifice. In general, too, the pulse is more irregular and unequal, smaller and more intermittent, in the former than in the latter case. Moreover, it is specially and exclusively in the former that the peculiar vibratory fremitus, which Corvisart designated by the word *bruissement*, is perceptible along the course of the large arterial trunks, particularly of such as are near to the heart.

In the following passage, the reader is informed how he may diagnosticate simple and uncomplicated *Hypertrophy of the Cardiac Valves*—a rather uncommon state of things, we ween.

" When the valves are the seat of a well-marked hypertrophic thickening, while at the same time they are exempt from malformation and can play freely, the valvular sounds are stronger, more sonorous than in a normal state, and resemble the clacking of a strong piston, or, better still, that produced by two sheets of parchment being applied smartly one against the other; hence the name *parchment sound, parchment clacking,* which I have given to the valvular sounds thus modified. In some persons, the attentive application of the fingers over the region corresponding to the hypertrophied, *parcheminés,* aortic valves, has made the action of these valves to be distinctly felt, and I regret not having sooner employed this mode of examination. In a good many cases already, trusting to the presence of the double phenomenon now stated, particularly of a well-marked parchment clacking, I have diagnosticated the existence of Hypertrophy of the Valves specified, and on several occasions dissection has confirmed the correctness of the diagnosis. This will surprise only those physicians who are little conversant with the subject on which we are engaged; the number of these is unhappily still too large."

We proceed to another form of cardiac lesion.

The *ramollissement* or softening of the muscular tissue *of the Heart* from inflammatory action (for M. Bouillaud expressly admits that this structural lesion may arise from a very different cause), and the suppuration that occasionally takes place in consequence of it, are thus described in the " Nosographie."

" In the *second* period of Carditis, softening of the heart is apt to supervene. Of this morbid change there are three principal species; the red, the white, and the yellow. Whatever be the species present, the softening of the heart is recognized by the following characters. The tissue of this organ has lost its natural firmness, so that it readily gives way to the pressure of the finger: it has become friable, and even fragile. In the *red* softening, the muscular substance often exhibits a violet or a brownish colour; it is sometimes accompanied with the infiltration of a certain quantity of altered blood, like the lees of wine, into the muscular interstices and underneath the investing membranes of the heart. At a more advanced period of the disease, the *red* softening becomes transformed into the *white* or *greyish* kind. It is then that complete suppuration is sometimes to be seen. In the same way as this kind of softening follows upon the red kind, so the *yellow* softening is a sort of transformation of the white. This third species appears to me to belong to chronic pericarditis. Laennec compared the yellowish tint of the morbid change in question to that of some dead leaves, as they fall from the tree.

"Suppuration of the heart is coincident with one or other of the species of softening which we have now mentioned. The purulent matter is either simply infiltrated, or it is collected into a focus, and then there will either be a solitary or multiple abscesses. These abscesses may find their way either to the inner or to the outer surface of the heart."—Vol. I. p. 424.

*Ulceration* of the parietes *of the Heart*, the result of inflammatory action, commences almost invariably in the endocardial membrane, and thus proceeds from within outwards. It is much more frequent in the left ventricle than in any other of the cavities. The ulcerations vary a good deal in different cases, in point of number, extent, and depth. Occasionally the ventricular walls are completely corroded at one part; and then perforation must inevitably ensue. In a few cases, the effect of cardiac ulceration has been to give rise to a tumour or bulging of the ventricular parietes, altogether similar to an aneurism of an artery;—in short, to a genuine *consecutive false aneurism* of the heart. Formed by the external layers of the muscular walls (sometimes by the pericardial covering alone, which then plays the part of the external coat of an artery), the aneurismal cyst will contain a more or less considerable quantity of laminated coagula. The size of such a cyst has been known to vary from that of a filbert to that of a common egg, and even to be very much larger, as in a case detailed by Professor Breschet.

Of 13 cases of Aneurism of the Heart, which have been analysed by M. Reynaud, 6 were seated at the apex of this organ, and the remaining 7 in other points of its periphery. The rupture of a cardiac aneurism is a rare event. When this frightful accident occurs, it usually happens previous to the formation of an aneurismal pouch, and when the ulceration has destroyed the deepest-seated layers of the cardiac parietes.

Ulceration and Rupture may be limited exclusively to certain *columnæ carneæ*, and more especially to some one or more of those belonging to the mitral valve.

The Diagnosis of Aneurism of the Heart must, in all cases, be merely conjectural. In none of the cases recorded, had its presence been so much as suspected.

With a few remarks on the subject of *Arteritis*, we shall close the present article.

As with the Heart, so it is with the larger arteries; inflammation of their lining membrane, or in other words *Endo-arteritis*, is, according to M. Bouillaud, of much more frequent occurrence than is generally imagined. "The cases," says he, "in which, upon dissection, various arteries—more especially the aorta, and the carotid, cerebral, subclavian, axillary, iliac and crural vessels—have exhibited morbid alterations of a distinctly inflammatory origin, are, I may say without exaggeration, almost innumerable. For some years, too, I have been able to announce, during the life of my patients, these alterations with a degree of precision which has not a little surprised those who have not paid due attention to the subject. They usually coincide with lesions of the same sort in the heart, and are developed at the same time, under the influence too of the same causes;—in the first rank of which must be placed *Rheumatic Disease.*"

The necroscopic appearances of *acute* Arteritis are these: redness of the internal surface, thickening, injection and softening of the arterial parietes, a pseudo-membranous or even a genuinely purulent secretion in the cavity of the vessel, and the presence too of a sanguineous coagulum, with the consequent more or less complete obstruction of its tube. Occasionally, superficial ulcerations or erosions of the lining membrarane are met with. Once or twice, pus has been found external to this membrane. It is because of the softening of the arterial walls that an inflamed vessel is apt to give way under the action of a ligature, as was first remarked by Dupuytren.*

The morbid effects of *chronic* Arteritis are thus described by our author.

"The vascular parietes become thickened and hypertrophied; the ulcerations become larger and deeper, and may end in perforations; or, at length, the arteries undergo a calcareous or cretaceous degeneration, which renders them friable. In some cases, this degeneration effects their internal membrane alone. The whitish or yellowish points, striæ, patches or spots, so often observed on the inner surface of arteries, are the commencements or rudiments of this sort of accidental ossification (*inchoamenta ossificationis,* to use the language of Morgagni), in the same manner as the milky spots on the pericardium and pleura, are those of the cartilaginous or ossiform laminæ so often met with after chronic pericarditis and pleurisy. However this may be, the furfuraceous, sandy, or pulverulent matter found on the inner surface of arterial tubes—in the form of points, scales, insulated spots or long lines—is removable, in whole or in part, with the nail or blunt edge of the scalpel. The laminæ, which are fibrous, fibro-cartilaginous, or calcareous, are on the other hand so adherent to the internal membrane that they cannot be detached; they are in some degree incorporated with it. Occasionally, the calcareous productions project and form a sort of stalactitic deposit on the inner surface, which is generally unequal and rugous at the same time. These productions are often observed to be situated not on, but beneath, the inner membrane of the artery."

The *symptoms,* most truly indicative of Arteritis, are those which denote a more or less complete obstruction to the course of the red blood along the course of the vessel or vessels. Thus, when the disease occurs in any of the extremities, the limb becomes chilly and benumbed, and eventually mortifies. This morbid condition is not unfrequently met with in aged people; hence the term *gangrena senilis,* that has been usually applied to it. It is not, however, confined to old age. Most writers have affirmed that severe pain along the course of the affected artery is very generally present in this species of arteritis. But this is far from being a constant symptom. M. Bouillaud attributes it, when present, to irritation

* Andral, in his *Præcis d'Anatomie Pathologique,* thus alludes to another morbid change of the arterial parietes. "The middle coat of the arteries may become atrophied, as well as hypertrophied. In the former case, the proper constituent tissue of the vessel becomes gradually more and more effaced, and it tends to return to the condition of mere cellular texture. In consequence of the attenuation of its parietes, the artery acquires the character and appearance of a vein; so that, when cut across, its sides fall together."

of the nervous filaments which accompany certain arteries, especially those of the extremities. It is not present in inflammations of internal and other arteries which are not provided with sensory nerves.

We have already said that Rheumatism is a frequent exciting *cause* of arteritis, as it is of Carditis. The existence of inflammation in the structures, immediately adjoining to arterial trunks, is probably another cause of these becoming involved in the same morbid action. This point in pathology has hitherto not received the attention which it deserves.

*Aorta.*—It is highly probable that this great trunk is very often the seat of inflammation, considering the frequency with which morbid deposits and other changes, the result of inflammatory action, are found to affect its tissue. "The redness, the thickening, the pseudo-membranes and the pus, the formation of sanguineous concretions, the softening, the ulceration, and all its consequences, subsequently the hypertrophy with dilatation or contraction of the tube of the vessel, the induration, the cretaceous or earthy degenerations, &c. are so many alterations which are induced by aortitis."

In a case of double Pleuro-pneumony (that on the right side was towards the apex, that on the left towards the basis, of the lungs), M. Bouillaud diagnosticated, he tells us, the existence of inflammation and the presence of a coagulum in the ascending aorta, in consequence of the following auscultatory symptoms: "towards the left edge of the sternum, above and within (sternad to) the left mamma, there was a very strong blowing sound, which extended up to nearly the clavicle; it was isochronous with the pulse, and did not prevent the click-clack of the heart being heard." Two days subsequently, it was found that "a blowing and rubbing sound continued to be heard over the origin of the aorta; this sound resembled somewhat that known by the term *piaulement*, and became in some degree stronger as it was traced up to the superior part of the sternum." The patient died four days afterwards; and, on dissection, the following appearances were discovered:—

"The aorta—from an inch above its cardiac extremity to where it gives off its large branches—where it was in contact with the right lung, hepatised at its apex, exhibited on its external surface a beautiful redness, which contrasted strongly with the white colour of its other portions. The internal membrane of the arch of the aorta presented a uniform rosy hue, which, on the aortic valves, assumed a bright red, not removable by washing, and which contrasted in a very marked degree with the whiteness of the lining membrane of the left ventricle, and also of the pulmonic valves.

"Commencing in the descending portion, and extending along the whole course of the ascending portion of the thoracic aorta, was a sanguineous coagulum, which became thicker and more and more organised as it approached the arch, where it was perfectly white, of the size of the small finger, and, in short, not unlike in colour and form the spinal marrow stript of its envelopes. It adhered to the walls of the aorta so strongly, as to require pretty strong traction to separate it. It became considerably larger, and was in some degree expanded where it entered the left ventricle, three-fourths of whose cavity were nearly filled with it, leaving only a narrow passage for the blood to pass along. This coagulum was reflected around the mitral valve, and insinuated itself into the (left) auricle, where it was partly black and partly white, and was less dense than the portion in the ventricle. In its passage through the auriculo-ventricular orifice, it was entwisted around the *chordæ tendineæ*, to which it closely adhered.

"The right ventricle also contained an enormous coagulum, which was dense, white and fibrinous, investing the tricuspid valve, and penetrating into the auricular cavity, which it filled almost entirely. The formation of this coagulum appeared to be more recent than that in the left cavities of the heart."

The diagnosis of a cretaceous degeneration of the aorta, with hypertrophy and dilatation of its parietes, is declared to be in many cases practicable, by attention to the following circumstances. "By inspection, palpitation, and percussion, the degree of dilatation and of the force of the arterial impulse may be exactly ascertained. At the same time, palpitation enables us to recognise, when the inner surface of the vessel is very rough and rugose, a very decided vibratory fremitus; and, by auscultation, we may generally hear over the region of the aorta a blowing sound, which is usually double, and sometimes so strong that I have compared it to the sound of a curry-comb (*bruit d'etrille).*"

We need scarcely repeat our author's opinion that the usual causes of acute Aortitis are rheumatism and the existence of inflammation of the lungs or pleura in the immediate contiguity of the vessel.

*Pulmonary Artery.*—On the subject of Inflammation of this vessel, M Bouillaud's remarks are to this effect:

"A case is related by M. Cruveilhier* of this disease occurring in a woman, who had suffered from uterine and crural Phlebitis after delivery. * * * The trunk and divisions of the pulmonary artery were filled with firm coagula. In the former, they were white and coherent; in the latter, they were of a red colour, and adhered but loosely to the walls of the vessel. In the centre of the principal clot was a deposit of phlegmonous pus. * * * There were traces of a lobular Pneumonia. An acute œdema was, in M. Cruveilhier's opinion, the result of this lesion. This idea is questionable; seeing that the pulmonary artery, although conveying venous blood, does not bring the blood back from an organ to the heart as proper veins do, but carries it to the lungs from the heart. I cannot therefore agree with M. Cruveilhier in attributing the pulmonary œdema to the obstruction of the pulmonary artery. I have met with a great many cases of recently-formed fibrinous coagula in the pulmonary artery, after severe Pneumonia, Pleurisy, &c."

The paper of Mr. Paget "On Obstructions of the Branches of the Pulmonary Artery," in a recent volume of the Medico-Chirurgical Transactions,† has directed the attention of physicians to a point in pathology which had previously been quite overlooked.

---

* *Anatomie Pathologique,* XI. Livraison.

† An ample analysis of Mr. Paget's Paper will be found in the Number of this Journal for April 1845.

